# Attitudes to ageing and objectively-measured sedentary and walking behaviour in older people: The Lothian Birth Cohort 1936

**DOI:** 10.1371/journal.pone.0197357

**Published:** 2018-05-16

**Authors:** Catharine R. Gale, Iva Čukić, Sebastien F. Chastin, Philippa M. Dall, Manon L. Dontje, Dawn A. Skelton, Ian J. Deary, for the Seniors USP Team

**Affiliations:** 1 Centre for Cognitive Ageing & Cognitive Epidemiology, Department of Psychology, University of Edinburgh, Edinburgh, United Kingdom; 2 MRC Lifecourse Epidemiology Unit, University of Southampton, Southampton, United Kingdom; 3 Institute for Applied Health Research, School of Health and Life Sciences, Glasgow Caledonian University, Glasgow, United Kingdom; 4 Department of Movement and Sports Sciences, Faculty of Medicine and Health Sciences, Ghent Univeristy, Ghent, Belgium; Nathan S Kline Institute, UNITED STATES

## Abstract

**Background:**

Prolonged sitting and low activity—both common in older people—are associated with increased mortality and poorer health. Whether having a more negative attitude to ageing is associated with higher levels of these behaviours is unclear.

**Objective:**

We investigated the prospective relationship between attitudes to ageing and objectively measured sedentary and walking behaviour.

**Methods:**

Participants were 271 members of the Lothian Birth Cohort 1936. At age 72 years, participants completed the Attitudes to Ageing Questionnaire which assesses attitudes on three domains—Psychosocial loss, Physical change and Psychological growth. At age 79 years, participants wore an activPAL activity monitor for seven days. The outcome measures were average daily time spent sedentary, number of sit-to-stand transitions, and step count.

**Results:**

There were no significant associations between any of the Attitude to Ageing domain scores and time spent sedentary or number of sit-to-stand transitions. In sex-adjusted analysis, having a more positive attitude to ageing as regards Physical change was associated with a slightly higher daily step count, for a SD increment in score, average daily step count was greater by 1.5% (95% CI 0.6%, 2.4%). On further adjustment for potential confounding factors these associations were no longer significant.

**Conclusion:**

We found no evidence that attitudes to ageing at age 72 were predictive of sedentary or walking behaviour seven years later. Future studies should examine whether attitudes to ageing are associated with objectively measured walking or sedentary behaviour at the same point in time. The existence of such an association could inform the development of interventions.

## Introduction

There is now considerable evidence that prolonged sitting can have adverse consequences for health. A recent systematic review and meta-analysis showed that sedentary time is associated with increased mortality and morbidity [1]. Physical activity is known to prevent premature death [2]. According to the review by Biswas et al, the increased health risk associated with sedentary behaviour appears to be independent of physical activity [1], although a subsequent systematic review suggested that the size of the mortality risk associated with sedentary time is reduced in those who are physically active and that the risk disappears in those who are most active, defined here as 60–75 minutes per day of moderate intensity activity [3]. There is a need to identify modifiable risk factors for sedentary behaviour [4, 5]. This may be particularly important for older people who spend a higher proportion of time sitting than younger age groups. In a systematic review, nearly 60% of those aged ≥60 reported sitting for ≥four hours a day, while in a survey where sedentary behaviour was measured objectively, 67% were sedentary for >8.5 hours per day [6].

One factor that might help determine both how sedentary and how physically active older people are is their attitude to ageing. There is evidence from cross-sectional surveys that older people who have a more negative attitude to ageing—seeing it as a time of ill health, loneliness, loss of ability to perform usual activities, and loss of independence—report lower levels of physical activity [7–10]. A limitation of these cross-sectional studies is that it is unclear whether attitudes to ageing influence activity patterns or *vice versa*. Longitudinal evidence on the relationship between attitudes to ageing and physical activity is sparse, but two studies have found that having a more positive view of ageing is associated with an increase in self-reported physical activity over time [10, 11]. In one of these studies, a positive attitude to ageing was predictive of walking more frequently six years later [10]. To our knowledge, there have been no longitudinal studies into the relation between attitudes to ageing and sedentary behaviour. Furthermore, existing longitudinal evidence on attitudes to ageing and physical activity is based solely on self-reported activity which could be subject to recall and social desirability bias. Establishing whether having a negative attitude to ageing is a risk factor for sedentary or walking behaviour in older people is important, as evidence from interventions suggest that such attitudes are modifiable [12], so could potentially lead to behaviour change.

We used data from the Lothian Birth Cohort 1936 to investigate the prospective relationship between attitudes to ageing at age 72 years and objectively measured sedentary behaviour and physical activity at age 79. Our aim was to examine whether having a more positive attitude to ageing was predictive of being less sedentary and taking more steps around seven years later.

## Materials and methods

### Participants

The Lothian Birth Cohort 1936 (LBC1936) was set up principally to study cognitive ageing [13, 14]. In total, 1,091 community-dwelling people were recruited at a mean age of about 70 years. This study uses data from Wave 2 and Wave 4, when participants were aged about 72 and 79 years, respectively. Consecutive participants in the Wave 4 survey were invited to take part in a sub-study on sedentary behaviour and physical activity until the target sample size of 300 was achieved. Using data on effect sizes found in previous studies of potential determinants of sedentary behaviour [5], we carried out a series of power calculations based on a 5% significance levels and 80% power, and estimated that a sample size of 300 would be sufficient to detect weak to moderate associations with the relatively common risk factors that we planned to investigate. Ethical approval was obtained from the Multi-Centre Ethics Committee for Scotland and Lothian Research Ethics Committee. Participants gave written informed consent.

### Measures

#### Objective measures of sedentary and walking behaviour

Sedentary behaviour and physical activity were measured using the activPAL monitor (activPAL3c, PAL Technologies Ltd, Glasgow, UK), which provides accurate and reliable measurements [15, 16]. The device is small and light (53x35x7mm; 15g) and was worn attached to the anterior thigh of the dominant leg with a waterproof dressing. Participants were asked to wear the activPAL continuously for seven days, including overnight and during bathing/swimming, while going about their usual daily activities. Participants kept a diary reporting the time they fell asleep the previous night and the time they woke up for each day of monitoring.

The outcome measures are the percentage of time spent sedentary, number of sit-to-stand transitions and number of steps, all averaged over the seven days.

#### Attitudes to ageing

At the time of the Wave 2 survey, participants completed the Attitudes to Ageing Questionnaire as a postal questionnaire [17]. This validated questionnaire consists of 24 items which cover three domains [18]. The Psychosocial loss subscale consists of negatively worded items about psychological or social losses, such as ‘Old age is a time of loneliness’. The Physical change subscale consists of positively worded items reflecting physical functioning and health, such as ‘My health is better than I expected for my age’. Two of the items in this subscale are explicitly about exercise: ‘It is important to take exercise at any age’, and ‘I keep as fit and active as possible by exercising’. The Psychological growth subscale consists of positively worded items reflecting ‘wisdom’ or ‘growth’, such as ‘As people get older they are better able to cope with life’. Each item is scored on a five-point Likert scale, ranging from ‘Strongly agree’ to ‘Strongly disagree’. Lower scores on the Psychosocial loss subscale and higher scores on the Physical change and Psychological growth subscales and lower scores on the Psychosocial loss subscale indicate more positive attitudes to ageing.

#### Covariates

We chose sex, depressive symptoms, chronic physical disease, body mass index (BMI), difficulties with activities of daily living, and education, all measured at Wave 2, as potential confounders of the relationship between attitudes to ageing and sedentary and walking behaviour on the basis of prior studies [7, 19–22]. Within the narrow age range of the sample, there was no association between age in days and either percent of waking time spent sedentary, average number of steps per day, or average number of sit-to-stand transitions, so it was not included as a potential confounder. Symptoms of depression were assessed using the depression subscale of the Hospital Anxiety and Scale (HADS-D) [23]. For the purposes of the current study, we calculated this subscale score based on six items only after omitting the item ‘I feel as if I’m slowed up’ to avoid potential construct overlap with the sedentary behaviour measures. Participants provided information during interview on whether they had been diagnosed with diabetes, stroke, cardiovascular disease, high blood pressure, arthritis, or cancer; we derived a variable for number of chronic physical diseases present. Height and weight were measured with a portable stadiometer and electronic scales, respectively. BMI was calculated as weight (in kilograms)/height (in metres)^2^. Participants completed the nine-item Townsend Disability scale that assesses difficulties with activities such as cutting toenails, washing, getting on a bus [24]. Participants also provided information on years of full-time education.

#### Statistical analysis

We used rank order correlations to examine percentage of time spent sedentary, number of sit-to-stand transitions, and step count in relation to other characteristics. These were used rather than Pearson correlations as many variables were either skewed or categorical. We used linear regression to examine the relationships between these outcome measures and scores on the attitude to ageing domains, controlling for the potential confounding variables. Average daily step count and average daily number of sit-to-stand transitions had a skewed distribution and were log transformed to give them a normal distribution. As we carried out a large number of tests of statistical significance, we controlled for multiple testing using the False Discovery Rate [25]. Analyses were carried out using Stata Statistical Software, release 13 (College Station, TX: StataCorp LP).

## Results

Of 374 people invited to participate, 304 were given an activPAL and 302 returned them. We excluded 31 participants (incomplete diary (n = 7), activPAL data quality (n = 5), and <7 days of data (n = 19)). We analysed only those who had 7 days of data so no assumptions about wear time would have to be made. Analyses are based on 271 participants. Mean age at the time of activPAL data collection was 79.1 (0.44) years.

Preliminary analyses showed that responses to the two items on exercise in the Attitude to Ageing Questionnaire were correlated with the activPAL measures collected around seven years later. People who endorsed the item ‘It is important to take exercise at any age’ more strongly spent less time sedentary (rho -0.174, p = 0.005), had a higher step count (rho = 0.325, p<0.001), and made more sit-to-stand transitions (rho = 0.235, p = 0.001). Similarly, people who endorsed the item ‘I keep as fit and active as possible by exercising’ more strongly spent less time sedentary (rho = -0.136, p = 0.027), had a higher step count (rho = 0.357, p<0.001), and made more sit-to-stand transitions (rho = 0.124, p = 0.045).

To avoid spurious correlations with the activPAL measures produced by construct overlap between measures, we calculated scores for the Physical change subscale after excluding the two items on exercise. Cronbach alpha statistics for Physical change, Psychosocial loss, and Psychological growth were 0.77, 0.82 and 0.75 respectively.

Table 1 describes the characteristics of the participants. Participants who spent a greater percentage of time sedentary tended to have a lower average daily step count (rho = -0.475, p<0.001), but sedentary time was not associated with number of sit-to-stand transitions. Participants having a higher average daily step count tended to make more sit-to-stand transitions (rho = 0.265, p<0.001). Participants who had a more positive attitude to psychosocial loss (indicated by lower scores) tended to make more sit-to-stand transitions (rho = -0.159, p<0.001); participants with more positive attitudes to physical change tended to have a higher daily step count (rho = 0.152, p<0.01). Scores on the domain of psychological growth were not associated with any activPAL measure. A higher percentage of sedentary time was associated with being female, having a higher BMI, and spending less time in full-time education. Taking more steps per day was associated with having fewer chronic physical diseases, lower levels of depressive symptoms, lower BMI, and lower scores on the Townsend disability scale. Making more sit-to-stand transitions per day was associated with lower BMI and lower scores on the Townsend disability scale.

**Table 1:** Characteristics of the participants at age 72 years and their rank order correlations with the sedentary and walking behaviour measures at age 79 years (n = 271).

Characteristics at age 72 years	Mean (SD), Median (IQR) or Number (%)	Correlation with % of time spent sedentary at age 79	Correlation with average number of steps per day at age 79	Correlation with average number of sit-to-stand transitions per day at age 79
Female, no (%)	131 (48.3)	-0.218**	-0.002	0.046
Attitude to psychosocial loss, median (IQR)	14 (11–17)	0.061	-0.097	-0.159***
Attitude to physical change, median (IQR)	20 (17–24)	-0.080	0.152**	0.073
Attitude to psychological growth, median (IQR)	28 (25–31)	0.007	0.082	0.032
BMI, mean (SD)	27.2 (4.14)	0.258***	-0.253***	-0.188**
Years of full-time education, mean (SD)	11.0 (1.21)	-0.133*	0.078	0.036
Number of chronic physical illnesses, median (IQR)	1 (1–2)	0.079	-0.178**	-0.109
HADS depression score, median (IQR)	1 (0–2)	0.103	-0.159**	-0.109
Townsend disability score, median (IQR)	0 (0–1)	0.078	-0.216**	-0.133*
**Objectively measured sedentary and walking behaviour at age 79 years**				
Percent of waking time spent sedentary, mean (SD)	62.5 (10.4)	-	-0.478***	-0.019
Average no. of steps per day, median (IQR)	6539 (4951–8761)	-0.478***	-	0.265***
Average no. of sit-to-stand transitions per day, median (IQR)	43.1 (35.3–51.4)	-0.019	0.265***	-

***p<0.001,

**p<0.01,

*p<0.05

We examined whether the relationships between attitudes to ageing and the activPAL measures varied by sex. Of nine interactions examined, only one was statistically significant (p = 0.028), but after control for multiple testing [25], this p value did not reach statistical significance (p = 0.25). We therefore analysed men and women together and adjusted for sex.

Table 2 shows regression coefficients (95% confidence intervals) for percentage of time spent sedentary, logged average daily step count, and logged average daily sit-to-stand transitions separately for a standard deviation (SD) increment in each attitude to ageing domain score. Results are shown adjusted first for sex, and then further adjusted for the potential confounding factors—education, depressive symptoms, number of chronic physical diseases, BMI, and Townsend disability score.

**Table 2:** Results from linear regression analyses of measures of sedentary behaviour at age 79 according to scores on attitudes to ageing scales at age 72.

	Regression coefficient (95% CI)		
Attitudes to Ageing scales, per point increase	Percent of time spent sedentary	Logged average number of steps per day	Logged average number of sit-to-stand transitions per day
Attitude to psychosocial loss			
Adjusted for sex	-0.057 (-0.252, 0.139)	-0.008 (-0.017, 0.002)	-0.007 (-0.013, -0.001)*^2^
Multivariable-adjusted^1^	0.015 (-0.226, 0.257)	-0.001 (-0.010, 0.009)	-0.005 (-0.012, 0.001)
Attitude to physical change			
Adjusted for sex	-0.138 (-0.362, 0.087)	0.015 (0.006, 0.024)**	0.003 (-0.003, 0.009)
Multivariable-adjusted^1^	-0.012 (-0.243, 0.219)	0.0001 (-0.006, 0.006)	0.001 (-0.006, 0.007)
Attitude to psychological growth			
Adjusted for sex	0.094 (-0.138, 0.325)	0.008 (-0.0001, 0.016)	0.001 (-0.004, 0.006)
Multivariable-adjusted^1^	0.047 (-0.241, 0.147)	0.005 (-0.003, 0.013)	-0.0002 (-0.005, 0.005)

^1^Adjusted for sex, HADS depression score, number of chronic physical illnesses, Townsend disability scale, BMI, and years of full-time education

^2^This association did not reach statistical significance after correction for multiple testing

***p<0.001,

**p<0.01,

*p<0.05

There were no associations between any of the attitude to ageing domains and percentage of waking time spent sedentary. There were no associations between attitudes to psychosocial loss or psychological growth and logged average daily step count. In the sex-adjusted model, participants who had a more positive attitude with regard to physical change took significantly more steps per day: for a SD increment in score on this domain, average daily step count increased by 1.5% (95% CI 0.6%, 2.4%). This remained significant after correction for multiple testing (p = 0.012) [25]. However, this relationship was no longer significant after adjustment for the other covariates, in particular depressive symptoms, BMI and Townsend disability score. In the sex-adjusted model, there was a significant association between having a more negative attitude to psychosocial loss and logged average daily sit-to-stand transitions. This association did not survive control for multiple testing (p = 0.10). It was attenuated after adjustment for the other covariates. There were no associations between the other attitudes to ageing subscales and logged average daily sit-t0-stand transitions.

As noted in the methods, we chose to exclude two items on exercise from the 8-item physical change domain to avoid spurious correlations with the activity measures. Had we retained the two items on exercise in the physical change domain, the associations between scores on this domain and amount of time spent sedentary or number of sit-to-stand transitions would have been very similar to those found when they were excluded. With the retention of these exercise items, the association between scores on this domain and average daily step count would have been slightly stronger and would have remained significant after adjustment for potential confounders: for a SD increment in score, average daily step count increased by 1.59% (0.86%, 2.31%) after adjustment for sex, and by 0.89% (0.14%, 1.64%) (p = 0.020) after further adjustment for potential confounding variables. However, this association was not statistically significant after correction for multiple testing (p = 0.12).

## Discussion

In this longitudinal study, we found no evidence that having a more positive attitude to ageing at age 72 was predictive of daily time spent sedentary or sit-to-stand transitions at age 79. In sex-adjusted analyses, people who had a more positive attitude with regard to physical change had a higher average daily step count, but the effect size was small and was attenuated to non-significance after adjustment for potential confounding factors.

Negative stereotypes of ageing are common [26]. Such internalised stereotypes may have adverse consequences for health in older people [27, 28]. Having a more negative perception of ageing has been associated with declines in cognitive ability [29] and in physical function [30, 31] and with an increased risk of dying prematurely [22, 32, 33]. Qualitative evidence in older women suggests that it is common for them to feel encouraged by society, family, and friends to sit rather than be physically active [19]. Although there is some longitudinal evidence to suggest that people who have a more positive attitude to ageing may be more physically active, as based on self-reports [10, 11], the results of the current study fail to confirm this. They also suggest that sedentary behaviour is unlikely to be a mechanism underlying the associations found in other studies between attitudes to ageing and adverse health outcomes.

According to the social psychologists, Ajzen and Fishbein, attitudes are more likely to predict behaviours if there is a strong degree of correspondence between attitude and behaviour such that both involve the same action, target, context and time element [34], in other words, both are measured at equivalent levels of specificity. There is considerable evidence in support of this principle of correspondence [35]. If we had collected data on participants’ attitudes to being physically active or sedentary, or on their attitudes to these same behaviours seven years in the future, we might have obtained stronger associations with our objective measures of walking and sedentary behaviour than those we found with the attitudes to ageing subscales which represent broader constructs.

Our findings are in contrast with those of two longitudinal studies which reported that a positive attitude to ageing was associated with increases in self-reported physical activity in older people over periods of 2.5 and 6 years respectively [10, 11]. In one of these studies, attitude to ageing was assessed with a 4-item scale about ageing as a time of personal growth and development [10], similar in content to the psychological growth subscale used here. The other study used the 5-item attitudes towards own ageing subscale of the Philadelphia Geriatric Center Morale Scale [11]. This includes items similar in wording to some of those included in the psychosocial loss and physical changes subscales used here, but has been criticized for ignoring the fact that ageing can be a time of development and positive change [18]. Neither of the attitudes to ageing scales used in these two longitudinal studies included items about physical activity [10, 11], yet despite what might seem a lack of correspondence between the attitude being assessed and the behaviour under study, they were significantly associated. One explanation for the inconsistency between these findings and those of the current study might be our use of objective measures of activity and sedentary behaviour rather than self-report.

Responses to two items in the Attitudes to Ageing Questionnaire that referred specifically to exercise—‘I keep as fit and active as possible by exercising’ and ‘It is important to take exercise at any age’—were significantly correlated in the expected direction with the objective measures of sedentary and walking behaviour made seven years later, confirming that self-reports of exercise have some validity. We chose to exclude these two items when calculating scores on the 8-item Physical change domain of the Attitudes to Ageing Questionnaire in order to avoid spurious correlations between these scores and the objective measures of sedentary and walking behaviour produced by the inclusion of these items on exercise. It might perhaps be argued that they should have been retained. The results from an analysis using all 8 items suggest that if we had included the two items on exercise in the physical change domain, our findings on the relationship between attitudes to physical change and objectively measured walking and sedentary behaviour seven years later would be essentially the same as those we obtained when they were excluded.

Our study has a number of strengths. The activPAL accelerometer provides gold-standard objective measures [15, 16]. Our analyses are based on accelerometry data for a seven day period thus minimising any systematic variation over the course of the week. Over 90% of participants provided a full week of data. Attitudes to ageing were assessed using a well-validated questionnaire [18]. A further strength is that we were able to take account of a range of potential confounding factors, including depressive symptoms, comorbidity, BMI and difficulties with activities of daily living. One potential weakness of our study is that attitudes to ageing were assessed on average seven years prior to the collection of activPAL data. However, a previous longitudinal study has shown that attitudes to ageing are predictive of frequency of self-reported walking six years later [10], so it was not unreasonable to hypothesise that in the current cohort we would find a similar association using objectively measured activity data. A further limitation is that we had no contemporaneous measure of sedentary and walking behaviour so were not able to examine whether attitudes to ageing were predictive of change in these behaviours.

In our ageing populations, understanding the extent to which internalised negative stereotypes about age affect health outcomes or behaviours in older people has particular relevance (28). In this study, we found no evidence that attitudes to ageing at age 72 years were predictive of objectively measured sedentary or walking behaviour around seven years later. Future studies should examine whether attitudes to ageing are associated with objectively measured walking or sedentary behaviour at the same time as the existence of such an association could inform the development of interventions, and investigate whether the relationship between attitudes to ageing and these outcomes varies over the wide age range of older adults. It might also be informative to explore whether specific attitudes about exercise or sitting in later life are better predictors of objectively measured walking or sedentary behaviour in older people than the broader-based attitudes to ageing scales.
